# Computer-directed rational engineering of dioxygenase TcsAB for triclosan biodegradation under cold conditions

**DOI:** 10.1128/aem.00346-25

**Published:** 2025-03-05

**Authors:** Yiran Yin, Xinjie Yu, Zongxin Tao, Christopher E. French, Zhenmei Lu

**Affiliations:** 1 MOE Laboratory of Biosystem Homeostasis and Protection, College of Life Sciences, Zhejiang University 98445 , Hangzhou, China; 2 Cancer Center, Zhejiang University 12377 , Hangzhou, China; 3 Zhejiang University-University of Edinburgh Joint Research Centre for Engineering Biology, International Campus, Zhejiang University 12377 , Haining, China; 4 School of Biological Sciences, University of Edinburgh 98275 , Edinburgh, United Kingdom; Danmarks Tekniske Universitet The Novo Nordisk Foundation Center for Biosustainability, Kgs. Lyngby, Denmark

**Keywords:** triclosan, dioxygenase, rational engineering, cold catalysis, biodegradation

## Abstract

The dioxygenase TcsAB is a specific dioxygenase involved in the initial biodegradation of the broad-spectrum antibacterial agent triclosan (TCS). However, it exhibits significantly reduced activity under cold conditions. In this study, a computer-directed approach combining loop engineering and N-terminal truncation was utilized to decrease the thermostability of TcsAB, thereby enhancing its catalytic activity in cold environments. The iterative mutant TcsAB (TcsA^Y277P/F279P/S311W/A313W^TcsB^N-terminal truncation^) exhibited a 2.54-fold greater catalytic efficiency than the wild type at 15°C. Molecular dynamics simulations showed that the mutations introduced in the substrate-binding pocket increased its flexibility, leading to enhanced catalytic activity through binding in a more advantageous conformation. This modified dioxygenase was employed as a biological component, and *Pseudomonas knackmussii* B13 was used as a chassis cell to construct an engineered strain for the efficient degradation of TCS at low temperatures. The objective was to enhance the capacity of TCS bioremediation in natural environments. Insights gained from this study may inform the rational redesign of enzymes related to the robustness of biodegradation of emerging contaminants.

**IMPORTANCE:**

The presence of TCS in surface water and wastewater poses a significant risk to aquatic organisms and human health due to its high resistance to degradation. The biodegradation of TCS pollution in the environment through the metabolic processes of microorganisms represents a significant and effective remediation strategy. The dioxygenase TcsAB is the only specific enzyme that has been identified as responsible for the initial biodegradation of TCS. Nevertheless, the enzyme activity responsible for the degradation of TCS was markedly diminished at low temperatures. The actual ambient temperature is frequently lower than the optimum temperature for enzyme reaction, and maintaining the 30°C reaction condition results in high costs and energy consumption for TCS removal. Accordingly, the rational engineering of dioxygenase TcsAB for low-temperature activity will facilitate more efficient and realistic removal of TCS in an aqueous environment.

## INTRODUCTION

In recent years, the discharge of emerging contaminants into aquatic environments has significantly impacted both ecosystems and human health. Triclosan (TCS), a synthetic antibacterial agent, is frequently detected in global aquatic systems (1). Although TCS concentrations in wastewater are generally low (approximately 17 ng/L–1.5 µg/L), higher concentrations have been found in activated sludge (0–50 mg/L) (2, 3). TCS is known to have bioaccumulative (4), endocrine-disrupting (5), and reproductive toxicity effects (6), posing threats to the health of aquatic organisms and humans (7). The rapid global spread of pandemic infectious illnesses has led to a sharp increase in the use of disinfectants and hand sanitizers containing TCS, which, in turn, has raised TCS levels in aquatic environments (8). As such, developing bioremediation technologies that can be integrated with current wastewater treatment processes is regarded as a cost-effective and promising strategy (2, 9).

Microbial biodegradation, particularly through enzyme-based systems, offers a promising approach to remediating TCS contamination. Microorganisms provide several advantages for wastewater treatment such as high activity, low costs, and reusability. Microbial and enzyme-based degradation strategies present cost-effective and eco-friendly solutions for remediating contaminated environments (10). The dioxygenase TcsAB of *Sphingopyxis* sp. MC1 is the only known specific enzyme capable of degrading TCS (11, 12). Previous studies have shown that TcsAB is involved in the initial biodegradation of TCS, converting it to 2,4-dichlorophenol (2,4-DCP) (12). In our earlier work, we used the chlorophenol-degrading strain *Pseudomonas knackmussii* B13 as the host cell, with *tcsAB* as the degrading functional component, to construct an engineered strain capable of efficiently degrading TCS (12). This engineered strain could rapidly degrade 10 mg/L TCS within 3 h at 30°C (12). However, since the ambient temperature during contaminant treatment often does not reach 30°C, maintaining such conditions results in high operational costs and energy consumption. Therefore, engineering the dioxygenase TcsAB to enhance its efficiency under lower temperature conditions holds significant theoretical and practical value for improving the remediation of emerging contaminants.

Increasing the catalytic activity of enzymes under low-temperature conditions is currently attracting increasing attention (13). Protein engineering, particularly through directed evolution, has proven to be a powerful tool for expanding the range of enzyme applications by altering their activity, stability, and selectivity (14). However, directed evolution requires extensive screening and a large number of iterations of mutagenesis, sequencing, and testing. Rational or semi-rational design approaches, guided by structural analysis, offer promising alternatives that can reduce the experimental workload, especially when straightforward screening methods for enzymatic reactions are not available (15). Despite these advances, significantly improving the catalytic efficiency of enzymes for low-temperature pollutant degradation remains a challenge. This is due to the limited understanding of the relationship between enzyme structure and function at low temperatures, particularly with respect to enzyme-substrate binding and interactions.

Loop regions that connect secondary structure elements (α-helices and β-strands) play a crucial role in protein function due to their increased interaction with solvents and ligands (16, 17). These loops, being the most flexible parts of enzymes, are key to protein evolution and the diversification of enzyme families. Sequence changes in these loop regions are often critical to altering enzyme function (18). Loop engineering strategies have shown considerable success in the rational and semi-rational design of enzymes, particularly through manipulating loops around substrate-binding pockets or channels. These modifications can influence critical parameters such as binding site volume and catalytic efficiency (19–21). Several studies have successfully used loop engineering to enhance the catalytic activity of enzymes at low temperatures by modifying the entrance to the substrate channel (22–25). However, there is still a lack of research on the rational engineering of enzymes specifically for the degradation of pollutants under low-temperature conditions.

In this study, we employed computer-aided methods to rationally engineer the TCS dioxygenase TcsAB to enhance its activity at low temperatures. The modified gene was heterologously expressed in *P. knackmussii* B13, increasing its ability to degrade TCS in cold environments. Our research not only highlights the importance of specific amino acid substitutions in enhancing enzyme activity at low temperatures but also provides a novel dioxygenase with enhanced degradation capabilities for emerging contaminants. This work lays a theoretical foundation for the subsequent use of engineered enzymes for bioremediation in environmental settings.

## RESULTS

### The enzymatic activity of TcsAB decreases under low-temperature conditions

**Fig 1:**
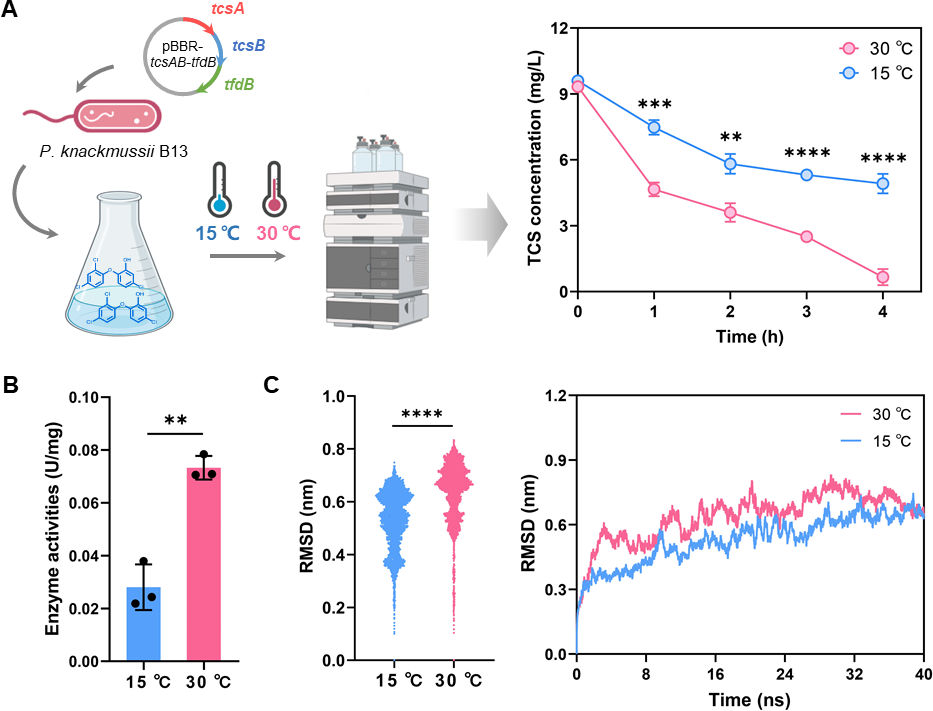
The impact of temperature on the activity of the TCS dioxygenase TcsAB. (**A**) Heterologous expression of the *tcsAB* gene cluster in *P. knackmussii* B13 under 15°C and 30°C conditions. (**B**) Measurement of TCS catalytic activity of purified TcsAB under 15°C and 30°C conditions. The data are presented as the means ± SDs, *n* = 3. Error bars represent the standard deviation calculated from three biological replicates. (**C and D**) The RMSD profile of TcsAB during 40 ns MD simulations under 15°C and 30°C conditions. **P < 0.01, ***P < 0.001, and ****P < 0.0001.

The engineered strain *P. knackmussii* B13-pBBR-*tcsAB-tfdB* showed a significant decrease in the efficiency of TCS degradation at 15°C compared to 30°C (Fig. 1A). Subsequently, the TCS dioxygenase TcsAB was purified separately (Fig. S1), and the purified TcsAB also showed significantly reduced TCS degradation activity at 15°C compared to 30°C (Fig. 1B), indicating a decrease in catalytic activity of TcsAB at low temperatures.

Under low-temperature conditions, the molecular movement of the enzyme is slower. A comparison of the molecular dynamics (MD) simulation of the TcsAB complex at 15°C and 30°C revealed that TcsAB exhibits enhanced stability and reduced mobility at the lower temperature (Fig. 1C). This is a contributing factor to the observed decline in enzymatic activity at 15°C.

### Structure-guided prediction of mutation sites of TcsAB

The protein structures of TcsA, TcsB, and the TcsAB complex were separately predicted using AlphaFold2 and AlphaFold3, and the accuracy of these predictions was evaluated. The accuracy of the TcsA structure predicted by AlphaFold2 was superior to that predicted by AlphaFold3, whereas the accuracy of the TcsB and TcsAB complex structures predicted by AlphaFold3 was more precise than that predicted by AlphaFold2 (Table S1). Moreover, the Ramachandran plot results for these protein structures demonstrated that the percentage of residues within the core area exceeded 90%, suggesting that the predicted structures are suitable for subsequent analysis (Table S1).

### Rational engineering of TcsA based on loop engineering and substrate-binding pocket reshaping strategy

**Fig 2:**
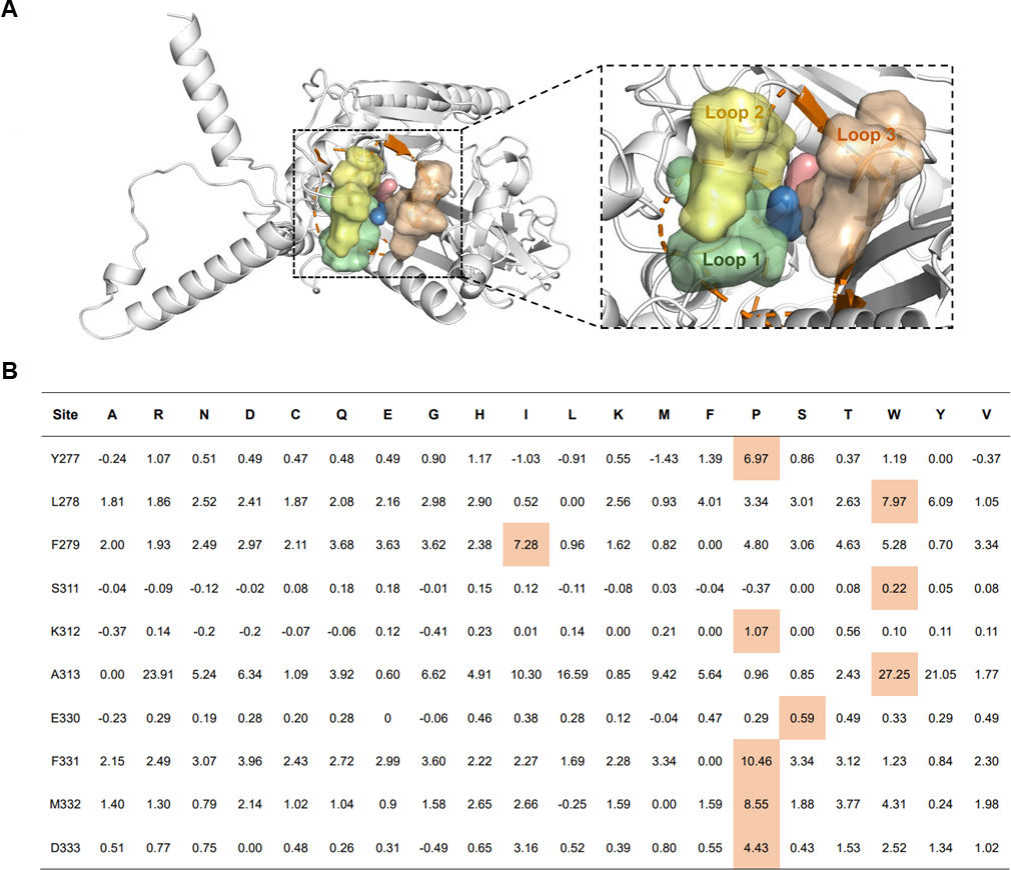
Rational engineering of TcsA to improve the catalytic activity of TcsAB under cold conditions. (**A**) Analysis of the loop regions for the substrate-binding pocket of TcsA. Loop1 (Tyr277, Leu278, and Phe279), loop2 (Ser311, Lys312, and Ala313), and loop3 (Glu330, Phe331, Met332, and Arg333) are shown in pale green, yellow, and brown, respectively. (**B**) Calculated ΔΔ*G* values of candidate mutants (colored boxes: the highest value of ΔΔ*G* for each site).

TcsA is the large subunit of the TCS dioxygenase TcsAB (12). A strategy was developed to enhance the catalytic activity of TcsA under low-temperature conditions (Fig. 2; Fig. S2). TcsA has two substrate channels (Fig. 2A) and a substrate-binding pocket with a volume of 1,640 Å^3^ (12). The entrances of the two substrate channels of TcsA contain the following amino acids: Leu278, Phe279, Ile314, Ile317, His327, Phe331, Phe341, and Leu376. Tyr277/Leu278/Phe279, Ser311/Lys312/Ala313, and Glu330/Phe331/Met332/Arg333 are located in loop 1 (Tyr277→Phe279), loop 2 (Ala313→Ser311), and loop 3 (Glu330→Arg333). These loops are situated at the entrance of the substrate-binding pocket of TcsA. It is postulated that these loop regions serve as a guiding element for substrate binding at the entrance of the substrate-binding pocket for the substrate tunnels (Fig. 2A). Consequently, these sites also function as potential modification sites to enhance the catalytic activity of TcsA in low-temperature environments. To further confirm the amino acid residues in TcsA that require mutagenesis, we employed PyMOL to mutate 10 amino acid residues in the 3 loop regions of TcsA to 19 different amino acids, generating a total of 190 mutants. Subsequently, the alterations in the folding Gibbs free energy of these mutants in comparison to the wild-type TcsA were calculated. Mutations that result in ΔΔ*G* > 0 were considered favorable, and the amino acid residue with the highest ΔΔ*G* value at each site was selected as the candidate mutant for TcsA engineering (Fig. 2B). Ten TcsAB mutants, including TcsA^Y277P^TcsB, TcsA^L278W^TcsB, TcsA^F279P^TcsB, TcsA^S311^TcsB, TcsA^K312P^TcsB, TcsA^A313W^TcsB, TcsA^E330P^TcsB, TcsA^F331P^TcsB, TcsA^M332P^TcsB, and TcsA^D333P^TcsB, were then constructed by PCR site-directed mutagenesis. Their enzymatic activity in oxidizing TCS at 15°C was then assessed. Among the 10 mutants, TcsA^Y277P^TcsB, TcsA^F279P^TcsB, TcsA^S311W^TcsB, and TcsA^A313W^TcsB exhibited augmented enzymatic activity in comparison to that of wild-type TcsAB (WT) (Fig. S3A). These four amino acid mutation sites may serve as promising candidates for the construction of composite mutants.

### Rational engineering of TcsB based on molecular dynamics simulation and B-factor calculation

**Fig 3:**
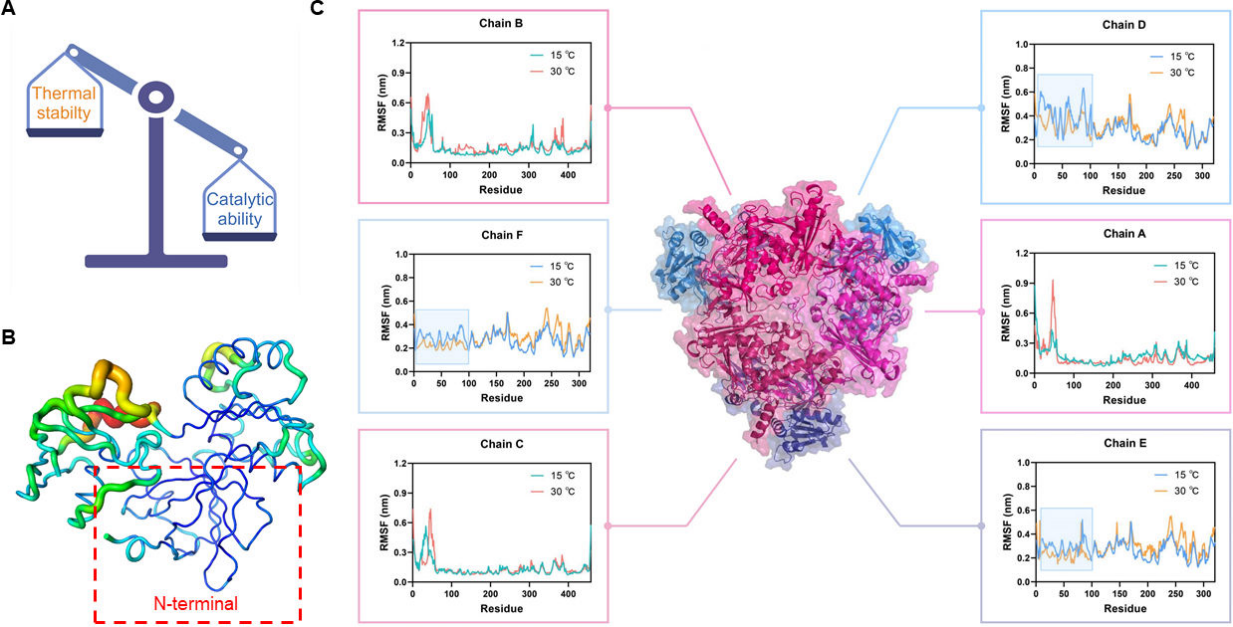
Rational engineering of TcsB to improve the catalytic activity of TcsAB under cold conditions. (**A**) The strategy of rational engineering for TcsB. (**B**) B-factor calculation of TcsB. (**C**) MD simulation of TcsAB complex under 15°C and 30°C conditions, respectively. Chains A, B, and C represent the three TcsA subunits, while chains D, E, and F represent those of TcsB.

Psychrophilic enzymes are defined by their high activity at low temperatures, which is often attributed to their relatively low thermal stability (13, 26). In this study, we aimed to enhance the flexibility of TcsB under low-temperature conditions by decreasing its thermal stability to improve its enzyme activity at low temperatures (Fig. 3A; Fig. S2). MD simulations and B-factor calculation revealed that the N-terminal region (2Thr-36Glu) of TcsB exhibited greater stability than other regions of the enzyme (Fig. 3B and C). Specifically, the stability of the TcsAB complex at low temperatures was evaluated using 40 ns MD simulations (Fig. 3C). The results demonstrate that the flexibility of the three TcsA chains in the α3β3 complex is not affected by temperature change, whereas the N-terminal regions (2Thr-36Glu) of the three TcsB chains exhibit reduced RMSF values at 15°C compared to 30°C, suggesting increased stability at low temperatures (Fig. 3C). These findings were further supported by the B-factor calculations, which showed consistent results (Fig. 3B). Accordingly, the mutant with wild-type TcsA and an N-terminal truncation of TcsB (TcsB-N) was constructed. The enzymatic activity of the WT and the TcsB-N in catalyzing TCS degradation under 15°C conditions was subsequently measured, and the enzymatic activity of the TcsB-N mutant was found to be slightly increased in comparison to that of the WT (Fig. S3B).

### The TcsAB iterative mutant enhances the catalytic activity for the degradation of TCS under low-temperature conditions

**Fig 4:**
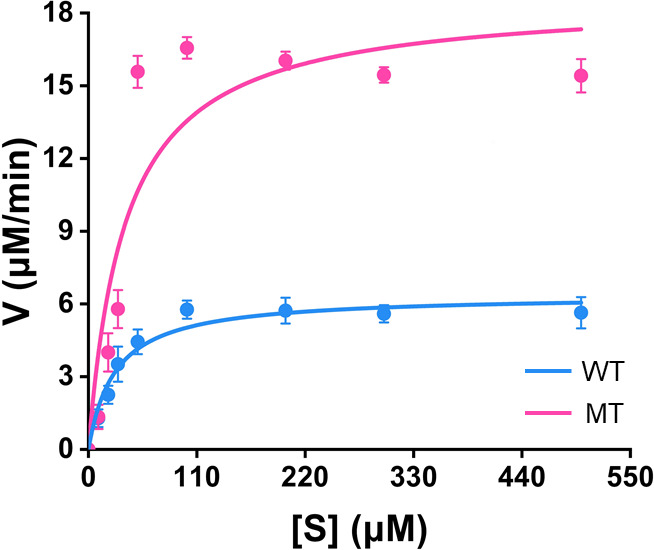
Enzymatic kinetics curve of WT and MT TcsAB in TCS oxidation at 15°C. The data are presented as the mean ± SD, *n* = 3. Error bars represent the standard deviation calculated from three biological replicates.

To identify superior mutants, iterative mutations were performed on four single-point mutants of TcsA (TcsA^Y277P^, TcsA^F279P^, TcsA^S311W^, and TcsA^A313W^) and the N-terminal truncation of TcsB. The enzyme-catalyzed reaction kinetics parameters of TCS degradation by the iterative mutant TcsA^Y277P/F279P/S311W/A313W^TcsB^N-terminal truncation^ (MT) at 15°C conditions were determined and compared with those of WT (Fig. 4; Table 1). The *k*_cat_ value of MT was 2.94-fold higher than that of WT (Table 1). The enzyme-catalytic efficiency (*k*_cat_/*K*_m_) exhibited a 2.54-fold increase under the assay conditions (Table 1). Thus, the rate of TCS degradation catalyzed by the mutant MT was faster than that by the WT at 15°C.

**TABLE 1:** Kinetic parameters of purified WT and MT TcsAB for TCS oxidation at 15°C

Type	*K*_m_ (μM)	*k*_cat_ (min^−1^)	*k*_cat_/*K*_m_
WT	35.33 ± 7.31	13.73	0.39
MT	40.56 ± 12.31	40.32	0.99

### Molecular mechanisms of the increased enzymatic activity of the TcsAB iterative mutant under low-temperature conditions based on molecular dynamics simulations

**Fig 5:**
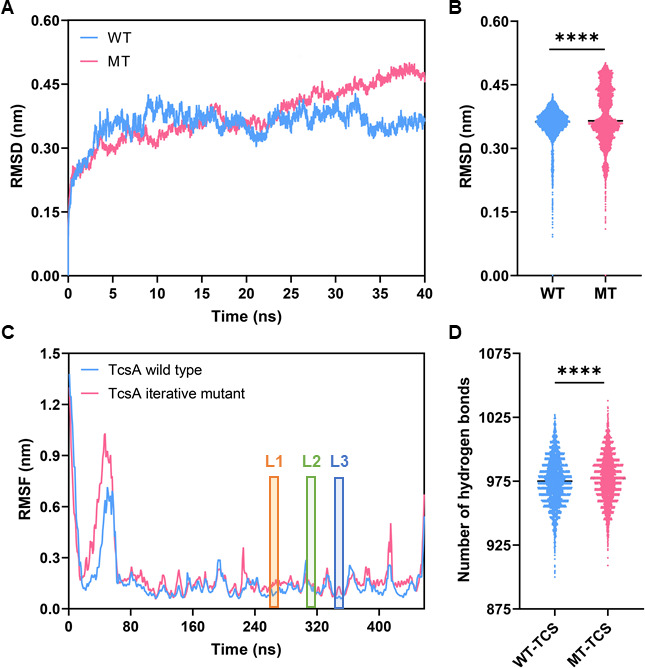
MD simulations elucidate the molecular mechanism responsible for the increased catalytic activity of rationally engineered TcsAB toward TCS at 15°C. The RMSD profiles (**A and B**) of WT and MT TcsAB during 40 ns MD simulations under 15°C conditions. (**C**) The RMSF profile of the wild-type TcsA and the TcsA iterative mutant during 40 ns MD simulations under 15°C conditions. L1: Loop1 (Tyr277, Leu278, and Phe279); L2: Loop2 (Ser311, Lys312, and Ala313); L3: Loop3 (Glu330, Phe331, Met332, and Arg333). (**D**) The number of hydrogen bonds of the wild-type TcsA and the TcsA iterative mutant bound to the substrate TCS. ****P < 0.0001.

To investigate the effect of temperature on enzyme kinetics, 40 ns MD simulations of the WT and MT TcsAB were performed at 15°C (Fig. 5). Throughout the entirety of the MD simulations, the root mean square deviation (RMSD) values of the MT were consistently higher than those of the WT, indicating that the structure of the MT exhibited greater flexibility at 15°C (Fig. 5A and B). These results all confirm that the TcsAB iterative mutant MT, derived from iterative mutations of four residues of TcsA and N-terminal truncation of TcsB, has a significantly beneficial impact on the overall structural flexibility compared to the WT. The root mean square fluctuation (RMSF) values of the wild-type TcsA and the TcsA iterative mutant (i.e., the TcsA^Y277P/F279P/S311W/A313W^ mutant) were then calculated. The RMSF values of the three loop regions at the substrate tunnel entrance in the TcsA iterative mutant were all greater than those in the wild type, indicating that the substrate channel loop regions of the TcsA iterative mutant became more flexible than those of the wild type (Fig. 5C). Models of the wild-type TcsA and the TcsA iterative mutant containing the substrate TCS were constructed (referred to as WT-TCS and MT-TCS, respectively) and the numbers of hydrogen bonds formed by the WT-TCS and the MT-TCS in the MD simulations were calculated. The MT-TCS complex was observed to form a greater number of hydrogen bonds, which is more conducive to the progression of the reaction (Fig. 5D). This also contributes to the higher degradation rate of TCS catalyzed by the MT compared to the WT under 15°C conditions.

### The heterologous expression of the TcsAB iterative mutant accelerates the degradation of TCS by *P*. *knackmussii* B13 under low-temperature conditions

**Fig 6:**
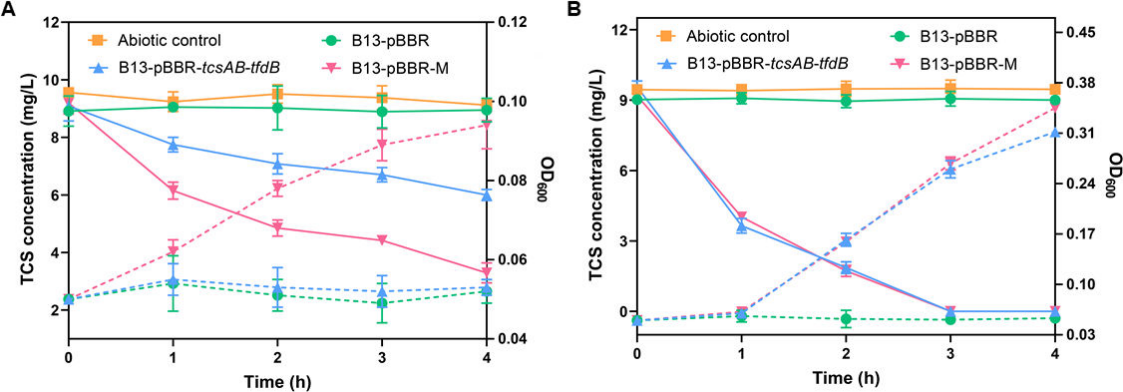
Heterologous expression of the mutant *tcsAB* in *P. knackmussii* B13 at 15°C (**A**) and 30°C (**B**). The solid line represents the concentration of TCS, while the dashed line indicates the OD_600_. The data are presented as the mean ± SD (*n* = 3). Error bars represent the standard deviation calculated from three biological replicates.

The mutant *tcsAB* was cloned into the expression plasmid pBBR1MCS2 to construct the expression plasmid pBBR-M, which was then heterologously expressed in *P. knackmussii* B13 to investigate its degradation of TCS under 15°C conditions (Fig. 6A). In comparison to *P. knackmussii* B13-pBBR-*tcsAB-tfdB*, the genetically modified strain *P. knackmussii* B13-pBBR-M, which expressed the cold-tolerant TCS dioxygenase gene *tcsAB*, exhibited a markedly elevated rate of TCS degradation at 15°C (Fig. 6A). At 30°C, the heterologous expression of the mutant gene *tcsAB* did not affect the biodegradation and utilization of TCS as a sole carbon source (Fig. 6B).

## DISCUSSION

The activity of functional microorganisms and enzymes in wastewater treatment plants (WWTPs) can be inhibited under cold conditions (27). Dioxygenases are widely involved in the degradation of emerging contaminants, but most enzymes are significantly less active at low temperatures than their optimal temperature range. From the perspective of contaminant remediation in the environment, the global average surface temperatures in 2022, 2023, and 2024 were 14.76°C, 15.08°C, and 15.19°C, respectively (28), which is below the optimal temperature for the majority of pollutant-degrading bacteria and enzymes involved in the degradation of pollutants. It is therefore of great significance to the field of bioremediation that dioxygenases with higher specific activity in low-temperature environments are developed. The use of a cold-active dioxygenase under actual environmental temperature conditions allows for the enhancement of contaminant remediation efficiency at actual environmental temperatures while saving energy costs.

Environmental remediation at temperatures close to ambient not only reduces energy consumption associated with heating but also minimizes side reactions, thereby reducing by-product formation (29, 30). Several studies have recently focused on addressing the challenge of low wastewater treatment efficiency under cold conditions. Many of these efforts involve screening cold-tolerant microorganisms for their potential in wastewater treatment applications. For example, Gao et al. (27) isolated a cold-tolerant denitrifying bacterium, *Achromobacter spiritinus* HS2, from an 8°C WWTP and demonstrated its ability to perform denitrification at low temperatures. In addition, some studies have enriched or constructed synthetic microbial communities for wastewater treatment under cold conditions. Wang et al. (31) enriched a psychrophilic bacterial community from a reactor, which was effective in removing nitrate and volatile fatty acids at 15°C. Another study focused on constructing a psychrophilic synthetic microbial community capable of efficiently degrading multiple pollutants in lab-scale reactors at 10–15°C (32). Furthermore, engineering enzymes to enhance their catalytic activity under low-temperature conditions holds substantial promise for improving their practical application. However, much of the current research on cold-active enzymes primarily focuses on the food industry, while fewer studies have explored their potential for environmental remediation.

Directed evolution is a well-established strategy in industrial enzyme engineering, where random mutations are induced in enzyme molecules through techniques such as error-prone PCR and gene recombination (33). Subsequently, high-throughput screening methods, which are designed to be precise, rational, and efficient, are employed to identify mutant enzymes with enhanced functional properties (34). Masahiko et al. (35) conducted error-prone PCR on the protease of the M4 family from the cold-adapted *Vibrio* sp. Pr21, resulting in the mutant Q301P showing increased activity and catalytic efficiency at both the optimal temperature of 40°C and under low-temperature conditions of 10°C. Zhao et al. (36) conducted directed evolution on the thermophilic alkaline serine protease from *Bacillus pumilus* BA06 to enhance its low temperature activity. The combinatorial mutant P9S/K27Q/T162I exhibited approximately fivefold higher casein hydrolysis activity compared to the wild-type enzyme at 15°C (36). The advantage of this approach is that the enzymatic activity can be modified without the need for extensive structure-function information on the protein, thus reducing the time required (34). However, this method entails a substantial and inefficient screening workload when there are no straightforward screening methods for mutant selection (37). In comparison with directed evolution, the rational design strategy requires a thorough understanding of the functional relationships within enzyme molecules, along with the prediction and analysis of their sequences and structures using computational tools (37, 38). This approach involves the strategic introduction of site-directed mutations into target enzymes through thoughtful design (38). Following the deciphering of the structure and catalytic mechanism of enzymes, rational design has become an efficient strategy to modify their catalytic properties. In this study, the structures of TcsA, TcsB, and the TcsAB complex were predicted by AlphaFold, and a rational design of TcsAB has been carried out. However, the actual protein structure of TcsAB has not been resolved yet, and there may still be deviations between the results of computational simulations and actual experimental results. Therefore, further analysis of the protein structure of TcsAB is still needed to more accurately guide rational engineering based on its structural information.

**Fig 7:**
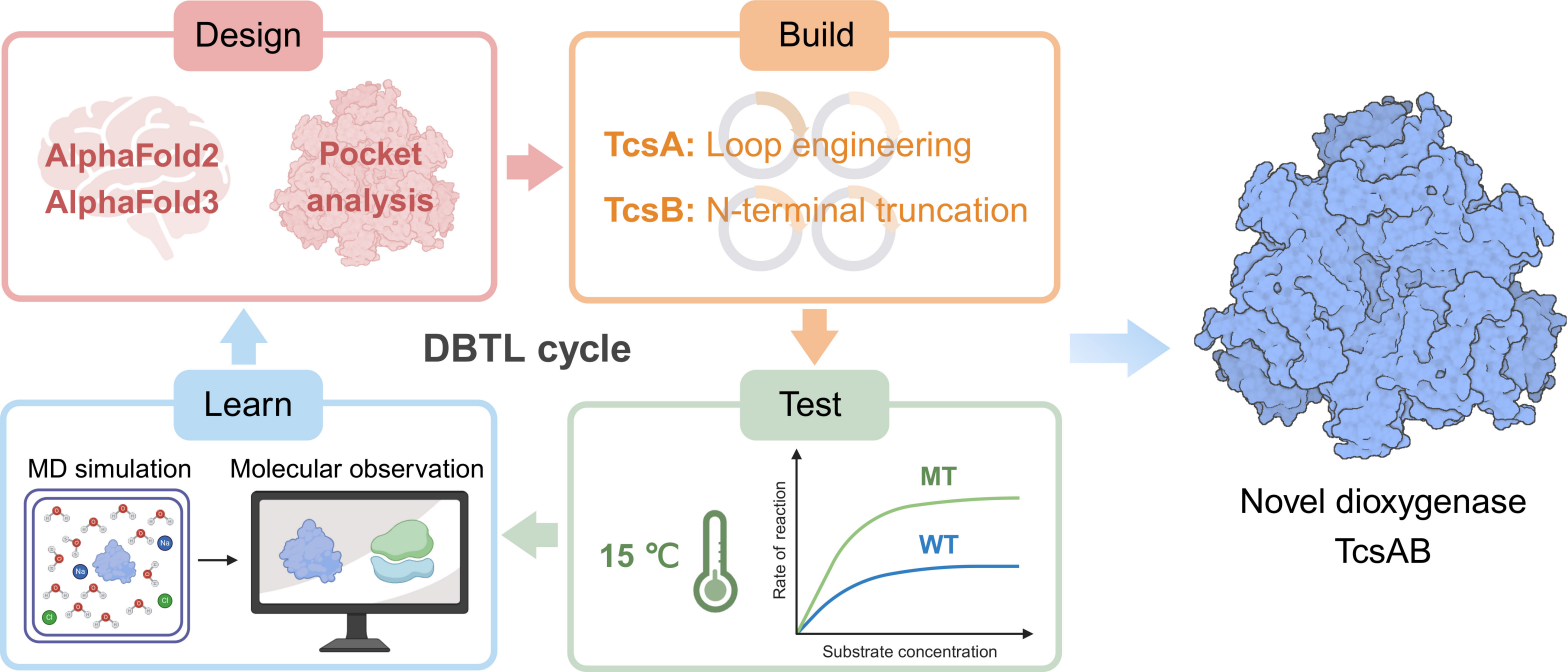
The DBTL cycle was employed in the rational engineering of TcsAB to enhance its catalytic efficiency under cold conditions.

Synthetic biology offers a methodology for engineering microorganisms capable of monitoring, aggregating, and degrading environmental pollutants (39). As the smallest functional units in synthetic biology systems, enzymes play a crucial role in degrading emerging contaminants. The mining of these catalytic mechanisms and their rational engineering have the potential to contribute to the bioremediation of emerging contaminants (39, 40). Accordingly, this study is based on the design-build-test-learn (DBTL) cycle of synthetic biology, employing methods such as computer-aided protein structure prediction, MD simulation, and Gibbs free energy calculation to design the dioxygenase TcsAB with improved catalytic activity for biodegrading the emerging contaminant TCS in low-temperature environments (Fig. 7). We incorporated this novel dioxygenase into engineered bacterial strains to efficiently degrade TCS at low temperatures, aiming to enhance bioremediation potential under ambient environmental conditions. This could be used to develop cold-tolerant TCS-degrading engineered strains. However, while the engineered strain *P. knackmussii* B13-pBBR-M demonstrates efficient TCS degrading at 30°C (Fig. 6B), its performance at 15°C still requires optimization (Fig. 6A). Future studies should explore synthetic biology approaches, such as screening cold-tolerant promoters and optimizing degradation pathways, to construct engineered strains with high TCS degradation efficiency under actual environmental conditions. Several studies have focused on TCS bioremediation, including the use of sequencing batch reactors and artificial wetlands (41–43), typically conducted at room temperature, which is below the normal growth temperature for microorganisms. Currently, no research has yet applied engineered bacteria for the biodegradation of emerging contaminants, especially for TCS, under low temperatures. This study represents a novel approach in this area. However, the application of engineered strains should be coupled with efforts to minimize environmental risks, such as the development of safety switch genetic circuits and post-release tracking (44).

Collectively, this study offers a novel perspective on designing and constructing a novel dioxygenase utilized in the bioremediation of an emerging contaminant. The application of this dioxygenase in TCS bioremediation has the potential for energy saving and improved environmental impact. Furthermore, it also provides theoretical guidance for efficient bioremediation of TCS at actual ambient temperatures. However, the precise crystal structure of TcsAB still requires further experimental elucidation, which will enhance the understanding of its functions and stability characteristics, thereby facilitating more targeted rational engineering.

## MATERIALS AND METHODS

### Chemicals, plasmids, strains, and cultivation conditions

TCS was purchased from Shanghai Yuanye Bio-Technology Co., Ltd. (Shanghai, China) and the stock solution was prepared at 10 g/L in methanol and sterilized through a 0.22 µm sterile filter before use. Isopropyl-β-D-thiogalactopyranoside (IPTG), nicotinamide adenine dinucleotide (reduced form) (NADH), and flavin mononucleotide (FMN) were purchased from Beyotime Biotech Inc. (Shanghai, China) (12). The compositions of lysogeny broth (LB) and minimal salt medium (MSM) were described in a previous study (45). The plasmids and bacterial strains used in this study are listed in Tables 2 and 3, respectively. *Escherichia coli* DH5α was used for vector construction, *E. coli* WM3064 was used for conjugation, and *E. coli* BL21(DE3) and *P. knackmussii* B13 were used as hosts for heterologous expression. *E. coli* strains were cultured in LB medium at 37°C, while *P. knackmussii* B13 was cultured in LB medium at 30°C. Kanamycin (Km) was used at a final concentration of 50  µg/mL. 2,6-diaminopimelic acid was used at a final concentration of 0.3  mM.

**TABLE 2:** Plasmids used in this study

Plasmid	Description*^a^*	Source
pETDuet	IPTG inducible coexpression vector, Amp^r^	Novagen
pETDuet-Km	Replace the ampicillin resistance gene of pETDuet with the kanamycin resistance gene.	This study
pETDuet-*tcsAB*	*tcsAB* fragment inserted into the pETDuet-Km linearized vector; Km^r^	This study
pETDuet-*tcsAB*-Y277P	pETDuet-*tcsAB* containing the TcsA^Y277P^ mutation; Km^r^	This study
pETDuet-*tcsAB*-L278P	pETDuet-*tcsAB* containing the TcsA^L278P^ mutation; Km^r^	This study
pETDuet-*tcsAB*-F279I	pETDuet-*tcsAB* containing the TcsA^F279I^ mutation; Km^r^	This study
pETDuet-*tcsAB*-S311W	pETDuet-*tcsAB* containing the TcsA^S311W^ mutation; Km^r^	This study
pETDuet-*tcsAB*-K312P	pETDuet-*tcsAB* containing the TcsA^K312P^ mutation; Km^r^	This study
pETDuet-*tcsAB*-A313W	pETDuet-*tcsAB* containing the TcsA^A313W^ mutation; Km^r^	This study
pETDuet-*tcsAB*-E330P	pETDuet-*tcsAB* containing the TcsA^E330P^ mutation; Km^r^	This study
pETDuet-*tcsAB*-F331P	pETDuet-*tcsAB* containing the TcsA^F331P^ mutation; Km^r^	This study
pETDuet-*tcsAB*-M332P	pETDuet-*tcsAB* containing the TcsA^M332P^ mutation; Km^r^	This study
pETDuet-*tcsAB*-D333P	pETDuet-*tcsAB* containing the TcsA^M332P^ mutation; Km^r^	This study
pETDuet-*tcsAB*-Y277P/F279P	pETDuet-*tcsAB* containing the TcsA^Y277P/F279P^ mutation; Km^r^	This study
pETDuet-*tcsAB*-Y277P/F279P/S311W/A313W	pETDuet-*tcsAB* containing the TcsA^Y277P/F279P/S311W/A313W^ mutation; Km^r^	This study
pETDuet-*tcsAB*-N	pETDuet-*tcsAB* containing the TcsB N-terminal truncation; Km^r^	This study
pET28a	Gene expression vector; Km^r^	Novagen
pET28a-*tcsA*	Vector for the synthesis and cloning of *tcsA*; Km^r^	(12)
pET28a-*tcsB*	Vector for the synthesis and cloning of *tcsB*; Km^r^	(12)
pET28a-His_6_-*tcsA*-His_6_	Vector for the expression and purification of TcsA; both the N-terminal and C-terminal of TcsA are labeled with 6 × His tags; Km^r^	This study
pET28a-His_6_-*tcsA*(Y277P/F279P/S311W/A313W)-His_6_	Vector for the expression and purification of the TcsA mutant; both the N-terminus and C-terminus of the TcsA mutant are labeled with 6 × His tags; Km^r^	This study
pColdII	IPTG and cold shock inducible vector; Amp^r^	(46)
pColdII-Km	Replace the ampicillin resistance gene of pColdII with the kanamycin resistance gene.	This study
pColdII-His_6_-*tcsB*	Vector for the expression and purification of TcsB; the N-terminus of TcsB is labeled with 6 × His tag; Km^r^	This study
pColdII-His_6_-*tcsB*-N	Vector for the expression and purification of TcsB N-terminal truncation; the N-terminus of TcsB is labeled with 6 × His tag; Km^r^	This study
pBBR1MCS2	Gene expression vector; Km^r^	(47)
pBBR-*tcsAB-tfdB*	Vector for the expression of the genes *tcsAB* and *tfdB* under the control of a *lac* promoter; Km^r^	(12)
pBBR-M	Vector for the expression of the genes *tcsA* mutant, *tcsB* mutant, and *tfdB* under the control of a *lac* promoter; Km^r^	This study

^*a*^ Amp^r^, ampicillin resistance; Km^r^, kanamycin resistance.

**TABLE 3:** Bacterial strains used in this study

Strain	Description*^a^*	Source
*E*. *coli* DH5α	*supE44 lacU169* (ϕ80d*lacZ* ΔM15) *recA1 endA1 hsdR17 thi-1 gyrA96 relA1*	Tsingke
*E*. *coli* WM3064	Donor strain for conjugation, DAP auxotroph	Laboratory stock
*E*. *coli* BL21(DE3)	Chaperonin gene *groESL* integrated into the genome	(48)
BL21-pETDuet-*tcsAB*	BL21(DE3) transformed with pETDuet-*tcsAB*; Km^r^	This study
BL21-pETDuet-*tcsAB*-Y277P	BL21(DE3) transformed with pETDuet-*tcsAB*-Y277P; Km^r^	This study
BL21-pETDuet-*tcsAB*-L278P	BL21(DE3) transformed with pETDuet-*tcsAB*-L278P; Km^r^	This study
BL21-pETDuet-*tcsAB*-F279I	BL21(DE3) transformed with pETDuet-*tcsAB*-F279I; Km^r^	This study
BL21-pETDuet-*tcsAB*-S311W	BL21(DE3) transformed with pETDuet-*tcsAB*-S311W; Km^r^	This study
BL21-pETDuet-*tcsAB*-K312P	BL21(DE3) transformed with pETDuet-*tcsAB*-K312P; Km^r^	This study
BL21-pETDuet-*tcsAB*-A313W	BL21(DE3) transformed with pETDuet-*tcsAB*-A313W; Km^r^	This study
BL21-pETDuet-*tcsAB*-E330P	BL21(DE3) transformed with pETDuet-*tcsAB*-E330P; Km^r^	This study
BL21-pETDuet-*tcsAB*-F331P	BL21(DE3) transformed with pETDuet-*tcsAB*-F331P; Km^r^	This study
BL21-pETDuet-*tcsAB*-M332P	BL21(DE3) transformed with pETDuet-*tcsAB*-M332P; Km^r^	This study
BL21-pETDuet-*tcsAB*-D333P	BL21(DE3) transformed with pETDuet-*tcsAB*-D333P; Km^r^	This study
BL21-pETDuet-*tcsAB*-Y277P/F279P	BL21(DE3) transformed with pETDuet-*tcsAB*-Y277P/F279P; Km^r^	This study
BL21-pETDuet-*tcsAB*-Y277P/F279P/S311W	BL21(DE3) transformed with pETDuet-*tcsAB*-Y277P/F279P/S311W; Km^r^	This study
BL21-pETDuet-*tcsAB*-Y277P/F279P/S311W/A313W	BL21(DE3) transformed with pETDuet-*tcsAB*-Y277P/F279P/S311W/A313W; Km^r^	This study
BL21-pETDuet-*tcsAB*-N	BL21(DE3) transformed with pETDuet-*tcsAB*-N; Km^r^	This study
BL21-pET28a-His_6_-*tcsA*-His_6_	BL21(DE3) transformed with pET28a-His_6_-*tcsA*-His_6_; Km^r^	This study
BL21-pET28a-His_6_-*tcsA*(Y277P/F279P/S311W/A313W)-His_6_	BL21(DE3) transformed with pET28a-His_6_-*tcsA*(Y277P/F279P/S311W/A313W)-His_6_; Km^r^	This study
BL21-pColdII-His_6_-*tcsB*	BL21(DE3) transformed with pColdII-His_6_-*tcsB*; Km^r^	This study
BL21-pColdII-His_6_-*tcsB*-N	BL21(DE3) transformed with pColdII-His_6_-*tcsB*-N; Km^r^	This study
*P*. *knackmussii* B13	Chlorophenol-degrading strain, used for heterologous gene expression; G^−^; Km^s^	(49)
B13-pBBR-*tcsAB-tfdB*	B13 transformed with pBBR-*tcsAB-tfdB*; Km^r^	(12)
B13-pBBR-M	B13 transformed with pBBR-M; Km^r^	This study

^*a*^ G^−^, gram-negative; Km^s^, kanamycin sensitivity; Km^r^, kanamycin resistance.

### Site-directed mutagenesis and production of TcsAB variants

The coding sequences of *tcsA* and *tcsB* were synthesized and codon-optimized from the genome of *Sphingopyxis* sp. MC1 (genomic accession number GCA_000371385.1) by Beijing Tsingke Biotech Co., Ltd. (Beijing, China) and Genscript Biotech (Nanjing, Jiangsu province, China) according to previously described methods (12). Site-directed mutagenesis of *tcsA* was carried out by PCR, as previously described, using the primers listed in Table S2. The plasmids pETDuet-*tcsAB* and pET28a-His_6_-*tcsA*-His_6_ were employed as a template for mutagenesis. Subsequently, the PCR products were transformed into *E. coli* DH5α competent cells and screened on LB agar containing Km. The resulting mutations were then confirmed by Sanger sequencing (Beijing Tsingke Biotech Co., Ltd., Beijing, China). The mutant plasmids were extracted and transformed into *E. coli* BL21(DE3) competent cells for subsequent experimentation.

### Purification of TcsAB and its variants

The PCR products of *tcsA* and *tcsB* were inserted into the multiple cloning site of the plasmids pET28a and pColdII using the ClonExpress II One Step Cloning Kit (Nanjing Vazyme Biotech Co., Ltd., Nanjing, Jiangsu province, China), generating the protein expression plasmids pET28a-His_6_-*tcsA*-His_6_ and pColdII-His_6_-*tcsB*, respectively. *E. coli* BL21(DE3) cells carrying the recombinant plasmids pET28a-His_6_-*tcsA*-His_6_ or pColdII-His_6_-*tcsB* were individually induced for heterologous expression. The specifics of heterologous expression conditions for TcsA and TcsB are detailed in Text S1. The cultured cells were harvested at 8,000 × *g* for 10 min and then resuspended in soluble protein binding buffer (20 mM Tris-HCl (pH 7.9), 10 mM imidazole, and 0.5 M NaCl). The collected cells were disrupted by sonication in an ice-water bath, followed by centrifugation at 11,000 × *g* for 45 min at 4°C to remove cell debris. The recombinant protein was then purified using Ni-nitrilotriacetic acid (NTA) agarose affinity chromatography (Jiangsu Cowin Biotech Co., Ltd., Taizhou, Jiangsu province, China) and eluted with soluble protein elution buffer (20 mM Tris-HCl (pH 7.9), 50–500 mM imidazole, and 0.5 M NaCl). The purified protein underwent desalting using BeyoDesalt G-25 Max Desalting Column (Beyotime Biotech Inc., Shanghai, China) and was then stored in desalting elution buffer (10 mM Tris-HCl (pH 7.4), and 50 mM NaCl). Protein concentrations were determined using a bicinchoninic acid (BCA) kit (KeyGen BioTECH, Nanjing, China). A sample of the protein was mixed with 5 × SDS loading buffer and analyzed by SDS-PAGE (12%) for identification.

### Enzyme assays and kinetic parameter measurements

*E. coli* BL21(DE3) carrying the expression vector pETDuet-*tcsAB* or the mutant plasmids was grown in LB medium at 37°C until it reached an OD_600_ of 0.6 and was then induced with 0.2 mM IPTG for 16 h at 16°C and 150 rpm. The reaction mixtures containing crude enzyme (21.3–36.4 μg) or purified enzyme (131.5–134.7 μg) were dissolved in 50 mM Tris-HCl (pH 8.0) and the reaction was initiated by the addition of 0.2 mM TCS. The enzymatic activities of crude or purified TcsAB and its mutants under 15°C conditions were monitored and calculated using established methods as described in previous research (12). One unit of enzyme activity (U) was defined as the amount of enzyme required to oxidize 1  µmol of electron donor (NADH) per minute. The purified TcsAB and the mutant were used to determine the kinetic parameters as described by Potrawfke et al. (50).

### Structure prediction and molecular dynamics simulation

The structures of monomer TcsA, TcsB, and TcsAB complex were predicted using AlphaFold2 (version 2.3.2) (https://github.com/google-deepmind/alphafold) (51) and AlphaFold3 (http://alphafoldserver.com/) (52). After the predicted structural models were obtained, SAVES (version 6.1) (https://saves.mbi.ucla.edu/) was used to evaluate the structures. The structures of the various mutants of TcsA, TcsB, and TcsAB complex were obtained by PyMOL (version 4.5) (http://www.pymol.org).

The Gibbs free energy of TcsA wild type (Δ*G*_WT_) and different TcsA mutants (Δ*G*_MT_) were calculated using YASARA (version 24.4.10) (https://www.yasara.org) (53) with the FoldX plug-in (http://foldxsuite.crg.eu) (54). The thermal stability of TcsA after mutation is calculated by Eq. 1.


(1) $$\Delta \Delta G=\Delta G_{MT}-\Delta G_{WT}$$

When ΔΔ*G* > 0, it indicates that the mutant is unstable compared to the wild-type enzyme; when ΔΔ*G* < 0, it indicates that the mutant is stable compared to the wild-type enzyme.

MD simulations were performed by employing GROMACS (version 2020.3) (55–57) using the AMBER99SB force field (55). The TcsAB complex was immersed in an octahedral box of TIP3P water. First, energy minimization, NVT at T = 303.15 K (30°C) or 288.15 K (15°C), and 100 ps NPT at 1 atm over a period of 100 ps were executed sequentially to equilibrate the simulation box (58, 59). Upon the completion of the two equilibration phases, a 40 ns MD simulation was run. The wild-type TcsAB complex, the mutant TcsAB complex, the complex formed between wild-type TcsA and TCS, and the complex formed between mutant TcsA and TCS were analyzed by MD simulation. RMSD, RMSF, and the number of hydrogen bonds were calculated and analyzed (55).

### Heterologous expression of *tcsAB* and its variant in *P*. *knackmussii* B13 under cold conditions

As previously described, the chlorocatechol-degrading “superstar” *P. knackmussii* B13 was selected as a heterologous expression host (12). The sequences of *tcsAB* and its mutants were synthesized based on the genome of *Sphingopyxis* sp. MC1 and the 2,4-DCP monooxygenase gene (*tfdB*) were synthesized based on the genome of *Sphingomonas* sp. YL-JM2C (genomic accession number GCA_000439355.1) (60, 61). The products were inserted into the pBBR1MCS2 linearized vector by homologous recombination using the ClonExpress MultiS One Step Cloning Kit (Nanjing Vazyme Biotech Co., Ltd., Nanjing, Jiangsu province, China). The plasmids pBBR-*tcsAB-tfdB* and the recombinant plasmid for the expression of the mutant genes *tcsAB* and *tfdB* (pBBR-M) were then transformed into *E. coli* WM3064-competent cells, which were subsequently conjugated with *P. knackmussii* B13. The recombinant strains were grown on 100  mL of MSM containing 10 mg/L TCS with an initial OD_600_ of 0.05 at 15°C and 200 rpm.

### Analytical methods

The concentrations of TCS were monitored by high-performance liquid chromatography (HPLC, Agilent 2000, USA) using an Eclipse XDB-C18 column (5 µm 4.6 × 250 mm). The aqueous samples (20 µL) were directly injected by an automatic injection. The culture samples were subjected to centrifugation at 5,000 × *g* for 10 min, after which the supernatant was collected and subjected to pretreatment with a 0.45 µm filter to remove the bacteria. The UV/Vis detector was set to 281 nm, and the HPLC mobile phase was 76% methanol and 24% H_2_O with a flow rate of 2.0 mL/min at 30°C.

### Statistical analysis

The protein structure models were visualized using PyMOL (version 4.5). Statistical analyses were performed using GraphPad Prism (version 8.4.2) (GraphPad Software Inc., San Diego, CA) and Origin Pro (version 2024b) (OriginLab Corporation, Northampton, MA). ChemDraw (version 19.0) (62) was used to determine the structural formulas of the chemical compounds. Images were created in https://BioRender.com/, with permission.

## SUPPLEMENTAL MATERIAL

Supplemental material aem.00346-25-s0001.docx Text S1, Figures S1 to S3, and Tables S1 and S2.

ASM does not own the copyrights to Supplemental Material that may be linked to, or accessed through, an article. The authors have granted ASM a non-exclusive, world-wide license to publish the Supplemental Material files. Please contact the corresponding author directly for reuse.
