# Supplementary material for: Computer-directed rational engineering of dioxygenase TcsAB for triclosan biodegradation under cold conditions
Source: Appl Environ Microbiol. 2025 Mar 5;91(4):e00346-25. doi: 10.1128/aem.00346-25 (PMC12016537; doi:10.1128/aem.00346-25)
Supplement: Supplemental material — Text S1, Figures S1 to S3, and Tables S1 and S2. [file aem.00346-25-s0001.docx]

**SUPPORTING INFORMATION**

**Title:** Computer-directed Rational Engineering of Dioxygenase TcsAB for Triclosan Biodegradation under Cold Conditions

**Running Title:** Rational Engineering of Dioxygenase TcsAB

Yiran Yin^1,2^, Xinjie Yu^1,2^, Zongxin Tao^1,2^, Christopher E. French^3,4^, Zhenmei Lu^1,2,3^ *

^1^MOE Laboratory of Biosystem Homeostasis and Protection, College of Life Sciences, Zhejiang University, Hangzhou 310058, China

^2^Cancer Center, Zhejiang University, Hangzhou 310058, China

^3^Zhejiang University-University of Edinburgh Joint Research Centre for Engineering Biology, International Campus, Zhejiang University, Hangzhou 310058, China

^4^School of Biological Sciences, University of Edinburgh, Edinburgh EH9 3FF, UK

*Address correspondence to Zhenmei Lu, [lzhenmei@zju.edu.cn](mailto:lzhenmei@zju.edu.cn)

**Content**

[**Supplemental Materials and methods** S3](#_Toc189730688)

[Text S1 S3](#_Toc189730689)

[**Supplemental Figures** S4](#_Toc189730690)

[Figure S1 S4](#_Toc189730691)

[Figure S2 S5](#_Toc189730692)

[Figure S3 S6](#_Toc189730693)

[**Supplemental Tables** S7](#_Toc189730694)

[Table S1 S7](#_Toc189730695)

[Table S2 S8](#_Toc189730696)

# Supplemental Materials and methods

Text S1**.** The heterologous expression conditions for TcsA and TcsB.

For the heterologous expression of TcsA protein, *E*. *coli* BL21(DE3) containing the recombinant vector pET28a-His_6_-*tcsA*-His6 should be grown to an OD_600_ of 0.6-0.8, followed by the addition of 0.2 mM IPTG to induce TcsA expression at 16℃ and 150 rpm for 14-16 h.

For heterologous expression of TcsB protein, *E*. *coli* BL21(DE3) containing the recombinant vector pColdII-His_6_-*tcsB* should be grown to an OD_600_ of 0.4-0.6, followed by rapid cooling in an ice bath for 30 min. The culture should then be induced with 0.2 mM IPTG for 18-20 h at 16℃ and 150 rpm for TcsB expression.

# Supplemental Figures


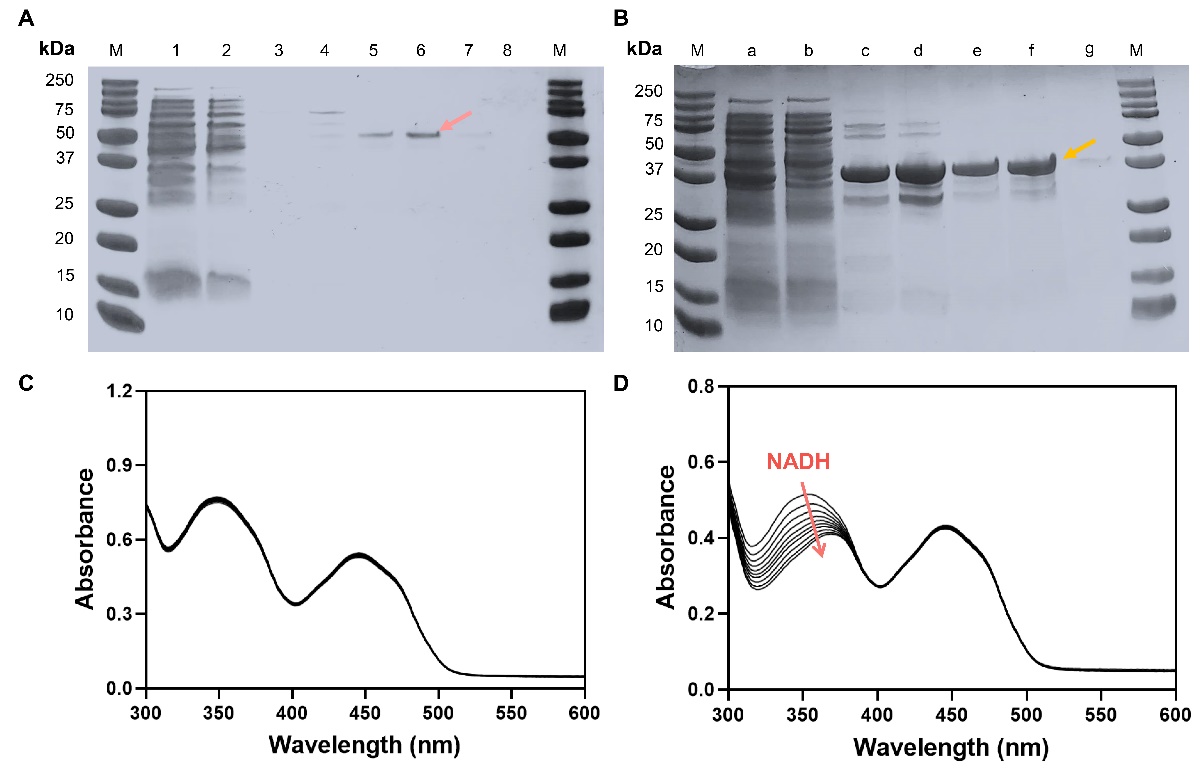


Figure S1**.** Purification of TcsAB and detection of the enzyme activity of the purified TcsAB towards the catalysis of TCS. (**A**) SDS-PAGE analysis of purified TcsA expression in the *E*. *coli* BL21(DE3) containing the recombinant vector pET28a-His_6_-*tcsA*-His_6_. Lane 1: Cell-free extracts of *E*. *coli* BL21(DE3)-pET28a-His_6_-*tcsA*-His_6_; Lane 2: Protein flow-through fluid; Lanes 3-7: Elution buffer containing 50-, 100-, 150-, 300-, and 500-mM imidazole, respectively ; Lane 8: Tris-HCl buffer (negative control). The pink arrow indicates purified TcsA. (**B**) SDS-PAGE analysis of purified TcsB expression in the *E*. *coli* BL21(DE3) containing the recombinant vector pColdII-His_6_-*tcsB*. Lane a: Cell-free extracts of *E*. *coli* BL21(DE3)-pColdII-His_6_-*tcsB*; Lane b: Protein flow-through fluid; Lanes c-g: Elution buffer containing 50-, 100-, 150-, 300-, and 500-mM imidazole, respectively. M: protein marker. The orange arrow indicates purified TcsB. (**C**) Spectra of reaction mixtures containing Tris-HCl buffer, TCS, NADH, FMN, Fe^2+^, and L-ascorbic acid (negative control). (**D**) Spectra of reaction mixtures containing purified TcsA, purified TcsB, NADH, FMN, Fe^2+^, and L-ascorbic acid. The red arrow indicates the directions of spectral changes of NADH.


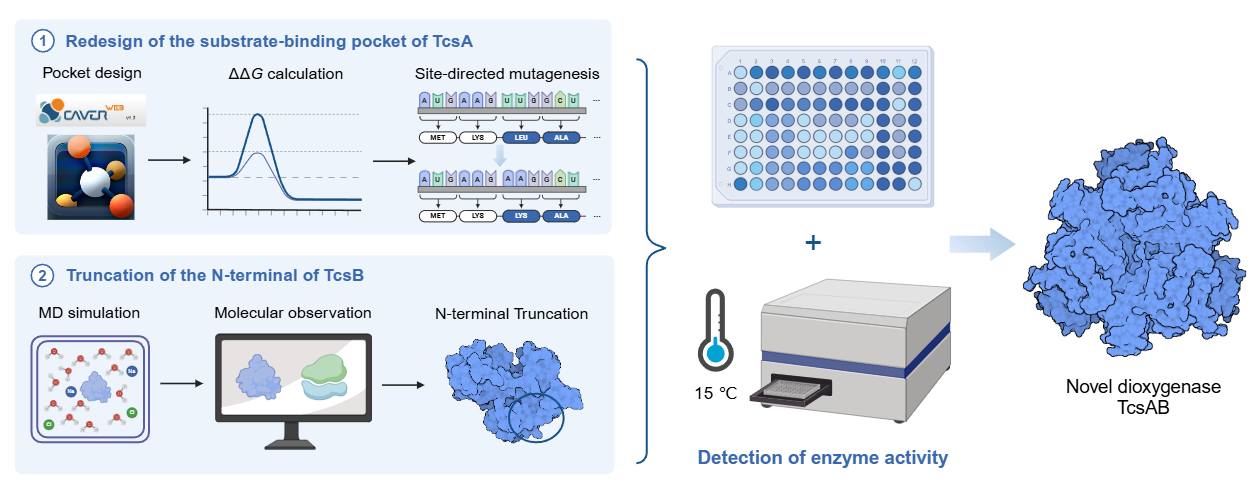


Figure S2**.** Schematic of the rational engineering workflow for improving the catalytic activity of TcsAB under cold conditions through stability calculation and MD simulation.


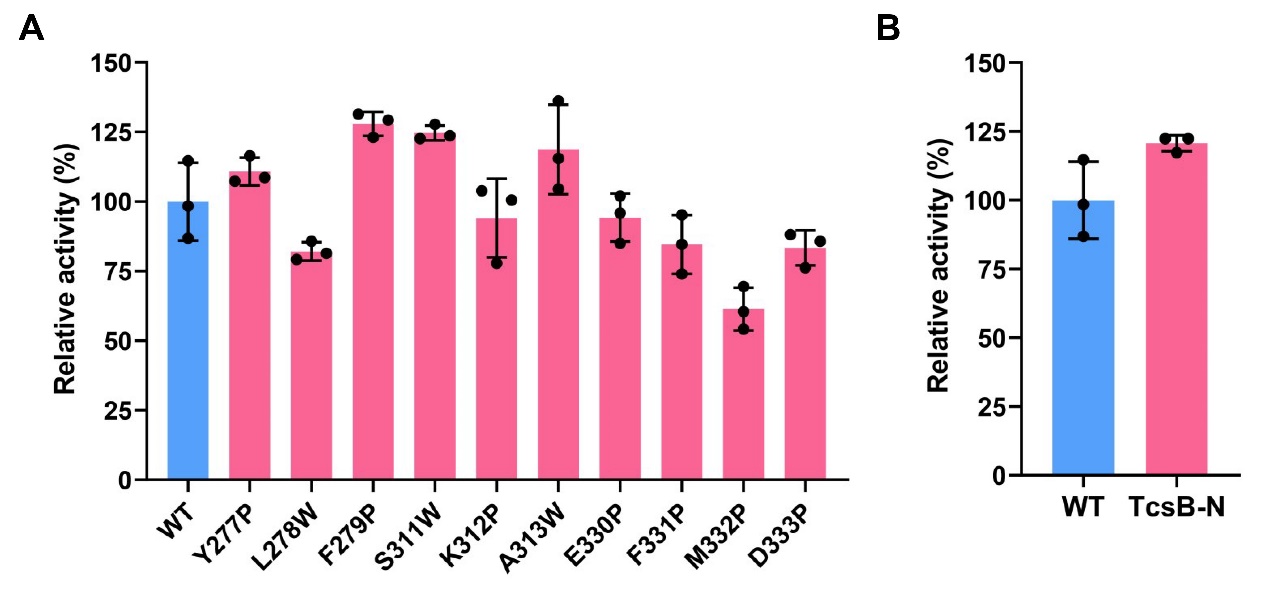


Figure S3**.** Relative enzyme activities of recombinant TcsAB and derivatives. (A) Enzyme activities of crude cell extract of *E*. *coli* BL21(DE3) containing TcsB with TcsA and various derivatives were determined at 15℃. WT: Wild-type TcsAB. The numbers represent the locations of the residue of TcsA that were modified while the alphabets are one letter code for amino acid. (B) TCS catalytic activity of crude cell extract of *E*. *coli* BL21(DE3) containing TcsA with wild-type TcsB (i.e., wild-type TcsAB, WT) and truncated TcsB (TcsB-N) at 15℃. The data are presented as the means ± SDs, n = 3. Error bars represent the standard deviations from three biological replicates.

# Supplemental Tables

Table S1**.** Evaluation for TcsA, TcsB, and TcsAB complex structures predicted using AlphaFold2 and AlphaFold3. a: The pLDDT values of the protein structures predicted by AlphaFold3 were calculated as the average pLDDT value of all the atoms in the protein. b: The numbers in this column represent the percentage of bases falling in the core area.

| **Protein** | **Software version** | **pLDDT ^a^** | **pTM** | **Ramachandran plot analysis ^b^** |
| --- | --- | --- | --- | --- |
| TcsA | AlphaFold2 | 88.3 | 0.89 | 91.7% |
|  | AlphaFold3 | 81.9 | 0.86 | 91.4% |
| TcsB | AlphaFold2 | 88.6 | 0.70 | 92.0% |
|  | AlphaFold3 | 91.4 | 0.91 | 92.3% |
| TcsAB | AlphaFold2 | 71.2 | 0.60 | 89.4% |
|  | AlphaFold3 | 78.9 | 0.63 | 90.1% |

Table S2**.** Primers used in this study. The red-highlighted nucleotide sequence represents the site of mutagenesis.

| **Primer** | **Sequence (5’–3’)** | **Purpose** |
| --- | --- | --- |
| pETDuet-L1-F | agggttattgtctcatgagc | Linearize the plasmid pETDuet |
| pETDuet-L1-R | ctgtcagaccaagtttactc |  |
| pETDuet-Km-F | aaacttggtctgacagttagaaaaactcatcgagca | Construct the plasmid pETDuet-Km |
| pETDuet-Km-R | atgagacaataacccttgtctcaaaatctctgatgt |  |
| pETDuet-V1-F | aggatcttcacctagatcc | Verify the plasmid pETDuet-Km |
| pETDuet-V1-F | agtggaacgaaaactcacg |  |
| pETDuet-L2-F | gaattcgagctcggcgcgcc | Linearize the plasmid pETDuet-Km |
| pETDuet-L2-R | ccatggtatatctccttcttaaagt |  |
| pETDuet-L3-F | gatatcggccggccacgcga |  |
| pETDuet-L3-R | catatgtatatctccttcttatacttaac |  |
| pETDuet-*tcsA*-F | aggagatataccatggatggcaacccgagccgacgac | Construct the pETDuet-*tcsAB* expression vector |
| pETDuet-*tcsA*-R | cgccgagctcgaattctcaagctgccggatagccga |  |
| pETDuet-*tcsB*-F | aaggagatatacatatgatgacaagaccatcaatacc |  |
| pETDuet-*tcsB*-R | gtggccggccgatatcttaaatgtccagcaccaggt |  |
| pETDuet-V2-F | agaggatcgagatcgatctc | Verify the pETDuet-*tcsAB* expression vector |
| pETDuet-V2-R | tcgattatgcggccgtgtac |  |
| pETDuet-V3-F | atcgtattgtacacggccgc |  |
| pETDuet-V3-R | tcagcggtggcagcagcctag |  |
| pETDuet-*tcsAB*-Y277P-F | acgtgcccgctgtttggtgaccagaaaatgg | Construct the pETDuet-*tcsAB*-Y277P site-directed mutagenesis vector |
| pETDuet-*tcsAB*- Y277P-R | aaacagcgggcacgtgcggtgcaggcagcca |  |
| pETDuet-*tcsAB*-L278P-F | gtgctactggtttggtgaccagaaaatgggcgg | Construct the pETDuet-*tcsAB*-L278P site-directed mutagenesis vector |
| pETDuet-*tcsAB*-L278P-R | accaaaccagtagcacgtgcggtgcaggcagccaac |  |
| pETDuet-*tcsAB*-F279I-F | ctacctgattggtgaccagaaaatgggcggcggcatc | Construct the pETDuet-*tcsAB*-F279I site-directed mutagenesis vector |
| pETDuet-*tcsAB*-F279I-R | ggtcaccaatcaggtagcacgtgcggtgcagg |  |
| pETDuet-*tcsAB*-S311W-F | gctggaaggcgatcgatgaaattcgttggttcatgcc | Construct the pETDuet-*tcsAB*-S311W site-directed mutagenesis vector |
| pETDuet-*tcsAB*-S311W-R | tcatcgatcgccttccagccgccatgaccaccggtttcg |  |
| pETDuet-*tcsAB*-K312P-F | gctctccggcgatcgatgaaattcgttggttcatgc | Construct the pETDuet-*tcsAB*-K312P site-directed mutagenesis vector |
| pETDuet-*tcsAB*-K312P-R | ttcatcgatcgccggagagccgccatgaccaccggtttcg |  |
| pETDuet-*tcsAB*-A313W-F | ggctctaagtggatcgatgaaattcgttggttcatgcc | Construct the pETDuet-*tcsAB*-A313W site-directed mutagenesis vector |
| pETDuet-*tcsAB*-A313W-R | atcgatccacttagagccgccatgaccaccggtttc |  |
| pETDuet-*tcsAB*-E330P-F | ggctctaagtggatcgatgaaattcgttggttcatgcc | Construct the pETDuet-*tcsAB*-E330P site-directed mutagenesis vector |
| pETDuet-*tcsAB*-E330P-R | atcgatccacttagagccgccatgaccaccggtttc |  |
| pETDuet-*tcsAB*-F331P-F | cgaagagccgatggatggcggggtgcacggtc | Construct the pETDuet-*tcsAB*-F331P site-directed mutagenesis vector |
| pETDuet-*tcsAB*-F331P-R | atccatcggctcttcgccatgatgcaccagg |  |
| pETDuet-*tcsAB*-M332P-F | aagagttccgggatggcggggtgcacggtctg | Construct the pETDuet-*tcsAB*-M332P site-directed mutagenesis vector |
| pETDuet-*tcsAB*-M332P-R | ccatccgggaactcttcgccatgatgcaccag |  |
| pETDuet-*tcsAB*-D333P-F | atggcgaaccgttcatggatggcggggtgcacg | Construct the pETDuet-*tcsAB*-D333P site-directed mutagenesis vector |
| pETDuet-*tcsAB*-D333P-R | catgaacggttcgccatgatgcaccaggttcgg |  |
| pETDuet-*tcsAB*-Y277P/F279P-F | tgccgggtgaccagaaaatgggcggcggcatc | Construct the pETDuet-*tcsAB*-Y277P/F279P site-directed mutagenesis vector |
| pETDuet-*tcsAB*-Y277P/F279P-R | tttctggtcaccccgcagcgggcacgtgcggtg |  |
| pETDuet-*tcsAB*-Y277P/F279P/S311W/A313W-F | gctggaagtggatcgatgaaattcgttggttcatgcc | Construct the pETDuet-*tcsAB*-Y277P/F279P/S311W/A313W site-directed mutagenesis vector |
| pETDuet-*tcsAB*-Y277P/F279P/S311W/A313W-R | tcatcgatccacttccagccgccatgaccaccggtttcg |  |
| pETDuet-V4-F | aagcagccatacggcactcc | Verify the site-directed mutagenesis vector |
| pETDuet-V4-F | tggcgactctcagcgcttgc |  |
| pETDuet-*tcsAB*-N-F | aggagatatacatatgatggcgggtagccacgtcga | Construct the pETDuet-*tcsAB*-N site-directed mutagenesis vector |
| pETDuet-*tcsAB*-N-R | ttctttaccagactcgttaaatgtccagcaccaggt |  |
| pET28a-L1-F | tgagatccggctgctaacaa | Linearize the plasmid pET28a |
| pET28a-L1-R | gtgatgatgatgatgatggctgctg |  |
| pET28a-His_6_-*tcsA*-His_6_-F | tcatcatcatcatcacgcaacaagggctgatgacga | Construct the pET28a-His_6_-*tcsA*-His_6_ recombinant vector |
| pET28a-His_6_-*tcsA*-His_6_-R | agcagccggatctcagtggtggtggtggtggtgtgccgccggataaccaacac |  |
| pET28a-His_6_-*tcsA*(Y277P/F279P/S311W/A313W)-His_6_-F | tcatcatcatcatcacgcaacaagggctgatgacg | Construct the pET28a-His_6_-*tcsA*(Y277P/F279P/S311W/A313W)-His_6_ recombinant vector |
| pET28a-His_6_-*tcsA*(Y277P/F279P/S311W/A313W)-His_6_-R | tggtggtggtggtgtgccgccggataaccaac |  |
| pET28a-V-F | acgatgcgtccggcgtagag | Verify the pET28a-His_6_-*tcsA*-His_6_ and pET28a-His_6_-*tcsA*(Y277P/F279P/S311W/A313W)-His_6_ recombinant vector |
| pET28a-V-R | cctcaagacccgtttagagg |  |
| pColdII-L1-F | cacatttccccgaaaagtgc | Linearize the plasmid pColdII |
| pColdII-L1-R | ctgtcagaccaagtttactc |  |
| pColdII-Kan-F | tttcggggaaatgtgttagaaaaactcatcgagca | Construct the pColdII-Kan recombinant vector |
| pColdII-Kan-R | aacttggtctgacagtgtctcaaaatctctgatgt |  |
| pColdII-V1-F | taccgcacagatgcgtaagg | Verify the pColdII-Kan recombinant vector |
| pColdII-V2-R | tcagtggaacgaaaactcac |  |
| pColdII-L2-F | tcgacctgcagtctagatagg | Linearize the plasmid pColdII-Kan |
| pColdII-L2-R | atgatgatgatgatgatgca |  |
| pColdII-His_6_-*tcsB*-F | tcatcatcatcatcatatgacaagaccatcaatacc | Construct the pColdII-His_6_-*tcsB* recombinant vector |
| pColdII-His_6_-*tcsB*-R | tctagactgcaggtcgattaaatgtccagcaccaggt |  |
| pColdII-V2-F | acgccatatcgccgaaagg | Verify the pColdII-His_6_-*tcsB* recombinant vector |
| pColdII-V2-R | ggcagggatcttagattctg |  |
| pBBR-L-F | gtgtgaaattgttatccgct | Linearize the plasmid pBBR1MCS2 |
| pBBR-L-R | cgaatggaaattgtaagcgt |  |
| pBBR-*tcsA*(Y277P/F279P/S311W/A313W)-F | tataggaacgaaaccatggcaacccgagccgacga | Construct the pBBR-M1 expression vector |
| pBBR-*tcsA*(Y277P/F279P/S311W/A313W)-R | tcaagctgccggatagccga |  |
| pBBR-*tcsB*-N-F | atccggcagcttgaatgggatcgcatgtcgatgtc |  |
| pBBR-*tcsB*-N-R | tcagatatcgagaaccagatcgg |  |
| pBBR-*tfdB*-F | ttacaatttccattcgtcagccggtcttcgcgatgg |  |
| pBBR-*tfdB*-R | gttctcgatatctgaatggcgaaaatcgttgaaacc |  |
| pBBR-F | ggatgtgctgcaaggcgatt | Verify the pBBR-M expression vector |
| pBBR-R | agttagctcactcattaggc |  |
